# The small heat shock protein B8 (HSPB8) efficiently removes aggregating species of dipeptides produced in C9ORF72-related neurodegenerative diseases

**DOI:** 10.1007/s12192-017-0806-9

**Published:** 2017-06-12

**Authors:** Riccardo Cristofani, Valeria Crippa, Giulia Vezzoli, Paola Rusmini, Mariarita Galbiati, Maria Elena Cicardi, Marco Meroni, Veronica Ferrari, Barbara Tedesco, Margherita Piccolella, Elio Messi, Serena Carra, Angelo Poletti

**Affiliations:** 1 Sezione di Biomedicina e Endocrinologia, Dipartimento di Scienze Farmacologiche e Biomolecolari (DiSFeB), Centro di Eccellenza sulle Malattie Neurodegenerative, Università degli Studi di Milano, Via Balzaretti 9, 20133 Milan, Italy; 2 C. Mondino National Neurological Institute, Pavia, Italy; 3 Dipartimento di Scienze Biomediche, Metaboliche e Neuroscienze, Università di Modena e Reggio Emilia, Modena, Italy; 4 Centro Interuniversitario sulle Malattie Neurodegenerative, Università degli Studi di Firenze Roma Tor Vergata, Genova e Milano, Italy

**Keywords:** RAN translation, Protein aggregation, Protein clearance, HSPB8, Motor neuron diseases

## Abstract

Amyotrophic lateral sclerosis (ALS) and frontotemporal dementia (FTD) are two neurodegenerative diseases in which similar pathogenic mechanisms are involved. Both diseases associate to the high propensity of specific misfolded proteins, like TDP-43 or FUS, to mislocalize and aggregate. This is partly due to their intrinsic biophysical properties and partly as a consequence of failure of the neuronal protein quality control (PQC) system. Several familial ALS/FTD cases are linked to an expansion of a repeated G4C2 hexanucleotide sequence present in the *C9ORF72* gene. The G4C2, which localizes in an untranslated region of the *C9ORF72* transcript, drives an unconventional repeat-associated ATG-independent translation. This leads to the synthesis of five different dipeptide repeat proteins (DPRs), which are not “classical” misfolded proteins, but generate aberrant aggregation-prone unfolded conformations poorly removed by the PQC system. The DPRs accumulate into p62/SQSTM1 and ubiquitin positive inclusions. Here, we analyzed the biochemical behavior of the five DPRs in immortalized motoneurons. Our data suggest that while the DPRs are mainly processed via autophagy, this system is unable to fully clear their aggregated forms, and thus they tend to accumulate in basal conditions. Overexpression of the small heat shock protein B8 (HSPB8), which facilitates the autophagy-mediated disposal of a large variety of classical misfolded aggregation-prone proteins, significantly decreased the accumulation of most DPR insoluble species. Thus, the induction of HSPB8 might represent a valid approach to decrease DPR-mediated toxicity and maintain motoneuron viability.

## Introduction

Amyotrophic lateral sclerosis (ALS) is a motoneuron disease (MND) in which upper motoneurons of the brain motor cortex and lower motoneurons of the bulbar region and of the anterior horn of the spinal cord are primarily, but not exclusively, affected. ALS may associate to other clinical conditions that diverge from pure MNDs and are typical features of other neurodegenerative diseases (NDs), like frontotemporal dementia (FTD). The presence of different mixed phenotypes (e.g., primarily MNDs with some FTD or vice versa) in ALS and FTD suggests similar pathogenic mechanisms (Ash et al. 2013; DeJesus-Hernandez et al. 2011; Ferrari et al. 2011; Renton et al. 2011). In fact, sporadic (sALS) or familial (fALS) forms of ALS exist, and some proteins mutated in fALS (like TAR DNA-binding protein 43 (TDP-43) and FUS RNA binding protein (FUS)) also have aberrant biochemical behaviors in sALS, even in their wild type (wt) forms. Some of these proteins are involved into pure familial (fFTD) or sporadic (sFTD) FTD or to mixed ALS/FTD forms (Robberecht and Philips 2013). Examples are mutant or wt TDP-43 or FUS, which tend to mislocalize from the nucleus to the cytoplasm, where they aggregate (Aulas and Vande Velde 2015; Mackenzie et al. 2010) and are hallmarks of ALS and FTD (Taylor et al. 2016). TDP-43 and FUS aggregations are due to their high propensity to misfold and to their poor removal via the neuronal protein quality control (PQC) system (Carra et al. 2012; Crippa et al. 2013a; Crippa et al. 2016b; Crippa et al. 2013b; Galbiati et al. 2014). Improved PQC system activity counteracts their aggregation in neuronal cells, thereby reducing toxic effect (Williams et al. 2006). Recently, several fALS and fFTD forms have been linked to an abnormal expansion of a hexanucleotide repeat of GGGGCC (G4C2) of the *C9ORF72* gene (DeJesus-Hernandez et al. 2011; Renton et al. 2011). The G4C2 transcript accumulates in nuclear RNA foci sequestering RNA-binding proteins (RBPs), thereby reducing their function (Peters et al. 2015; Rossi et al. 2015). At the same time, the expanded G4C2, which is located in the 5′-untranslated region of the C9ORF72 transcript, serves as template for an unconventional “repeat-associated ATG-independent” translation (RAN translation) (Ash et al. 2013; Cleary and Ranum 2013; Lashley et al. 2013; Mann et al. 2013; Mori et al. 2013a; Mori et al. 2013b). RAN translation of the expanded G4C2 (and its antisense C4G2) produces five different dipeptide repeat proteins (DPRs): poly Gly-Ala (polyGA), poly Gly-Pro (polyGP), poly Gly-Arg (polyGR), poly Pro-Arg (polyPR), and poly Pro-Ala (polyPA) (Mann et al. 2013; Mori et al. 2013a; Mori et al. 2013b). None of these dipeptides exist in normal individuals, and it is likely that DPRs exist in partially structured or metastable conformations. Indeed, they accumulate in inclusions in neurons and glia of ALS and/or FTD patients (Ash et al. 2013; Mann et al. 2013; Mori et al. 2013b). Interestingly, these DPR inclusions localize in the cytoplasm or in the nucleus of affected neurons (Freibaum and Taylor 2017), and sequester the SQSTM1/p62 autophagy receptor (Al-Sarraj et al. 2011), but are negative for TDP-43 even if the patients display a typical TDP-43 pathology (Freibaum and Taylor 2017). This suggests that they are identified by the PQC system for clearance, but the process may have failed. It is still highly debated which DPR is more toxic, and whether particular species may be responsible for neuronal alteration in ALS/FTD. A detailed analysis of the biochemical properties and the potential adverse effects of the single DPRs has been recently published by Freibaum and Taylor (2017). However, how these DPRs are recognized by and/or escape from the PQC system to aggregate is still unknown, and possibly, boosting the PQC might facilitate their removal, thereby reducing their toxicity.

Heat shock protein B8 (HSPB8) is a small heat shock protein (sHSP), which has been found mutated in specific forms of motor neurophaty (Ghaoui et al. 2016; Irobi et al. 2004; Tang et al. 2005), and it is highly expressed in anterior horn spinal cord motoneurons that survive in ALS mice at end stage of disease (Crippa et al. 2010a; Crippa et al. 2010b). HSPB8 has been shown to be particularly active in the removal of aggregating misfolded TDP-43 (Crippa et al. 2016a; Crippa et al. 2016b). HSPB8 acts as a chaperone and, in complex with Bcl-2 associated athanogene 3 (BAG3), HSP70 (and CHIP) recognizes misfolded TDP-43 species (Carra 2009; Carra et al. 2008b). Once bound to the HSPB8–BAG3–HSP70 complex, misfolded and aggregate-prone TDP-43 species are targeted to autophagic degradation (Crippa et al. 2010a; Crippa et al. 2010b). This process also requires an active dynein-mediated retrograde transport, which mediates the targeting of the bound cargo to the autophagosomes for clearance (Cristofani et al. 2017). The pro-degradative activity of HSPB8 is not limited to TDP-43, but it is exerted also on a number of other mutated proteins linked to neurodegenerative diseases. Examples include mutant SOD1 linked to some fALS (Crippa et al. 2010b); polyglutamine (polyQ) containing proteins like androgen receptor (ARpolyQ) and huntingtin (HTT) (Carra et al. 2008a; Giorgetti et al. 2015; Rusmini et al. 2013), causing spinal and bulbar muscular atrophy (SBMA) and Huntington’s disease (HD), respectively; beta-amyloid (A-beta) linked to Alzheimer disease (AD) (Wilhelmus et al. 2006); alpha-synuclein (alpha-syn) causing Parkinson’s disease (PD) (Bruinsma et al. 2011); and other misfolded proteins such as mutated HSPB5 causing neuropathy (Arndt et al. 2010; Chavez Zobel et al. 2003; Sanbe et al. 2009; Vicart et al. 1998). Collectively, these data demonstrate that, in mammalian cells, HSPB8 is able to recognize and interact with a large variety of misfolded protein conformations, avoiding their irreversible aggregation and promoting their autophagy-mediated disposal.

Here, we investigated whether the potent autophagy facilitator HSPB8 decreases the aggregation propensity of the five DPRs and decreases their accumulation, comparing its efficacy on the five different DPRs. Collectively, our data demonstrated that the activity of HSPB8 is not limited to classical misfolded proteins, but extends in cells to a large variety of aberrant peptides that may generate aggregating species.

## Methods

### Chemicals

The chemicals used were Z-Leu-Leu-Leu-al (MG132) (Sigma-Aldrich, C2211) and 3-methyladenine (3-MA) (Selleckchem, S2767).

### Plasmids and siRNA

The FLAG-tagged plasmids coding for FLAG-polyGA, FLAG-polyGP, FLAG-polyGR, FLAG-polyPR, and FLAG-polyPA were kindly provided by Prof. Daisuke Ito (Keio University School of Medicine). All plasmids code 100 repeats for each DPRs (Yamakawa et al. 2015). pCI-HSPB8 plasmid is routinely used in our laboratory and it has been previously described (Crippa et al. 2010b; Rusmini et al. 2013). pcDNA3 (Life Technologies, V790-20) plasmid was used to normalize for transfected plasmid DNA amount. pEGFPN1 (Clontech Lab, U55762) plasmid was used to evaluate transfection efficiency by fluorescent microscopy.

To silence endogenous HspB8 expression, we used a custom small interfering RNA (siRNA) duplex (*﻿HspB8﻿﻿* target sequence: CGG AAG AGC UGA UGG UAA AUU; non-target target sequence: UAG CGA CUA AAC ACA UCA AUU) (Dharmacon, Thermo Scientific Life Sciences).

### Cell cultures and transfection

The immortalized motoneuronal cell line NSC34 is routinely used in our laboratory (Crippa et al. 2010a, 2010b; Piccioni et al. 2002; Rusmini et al. 2011; Simeoni et al. 2000) and has been transfected with Lipofectamine (Life Technologies, 18324020)/transferrin (Sigma-Aldrich, T8158), as previously described (Crippa et al. 2016b; Giorgetti et al. 2015), using 0.6 μg of plasmid DNA, 4 μL of transferrin solution, and 2 μL of Lipofectamine (for well of 12-well plate). siRNA transfection was performed with Lipofectamine 2000 (Life Technologies, 11668019) using 40 pmol of target RNA and following manufacturer’s instructions.

### Microscopy analyses

NSC34 cells were plated in 24-well multiwell plates containing coverslips at 35,000 cells/well density, transiently transfected with the plasmid coding for FLAG-DPRs as previously described. Then, the cells were fixed and processed as previously described (Sau et al. 2007). The following primary antibodies were used to analyze protein distributions: mouse monoclonal ANTI-FLAG M2 (dilution 1:500; Sigma, F3165), homemade rabbit polyclonal anti-HSPB8 no. 3 (dilution 1:200), and homemade rabbit polyclonal anti-human HSPB8 no. 25 (dilution 1:200). All antibodies were diluted in TBS-T containing 5% nonfat dried milk powder (Euroclone, EMR180500). Secondary antibodies were as follows: Alexa Fluor 488 anti-mouse (dilution 1:1,000; Life Technologies, A11017) and Alexa Fluor 594 anti-rabbit (dilution 1:2,000; Life Technologies, A11072) in TBS-T containing 5% nonfat dried milk powder (Euroclone, EMR180500). Cells were stained with Hoechst to visualize the nuclei. Images were acquired with LSM510 Meta system confocal microscope (Zeiss, Oberkochen, Germany). Images were processed with the Aim 4.2 software (Zeiss).

### WB analysis and FRA

NSC34 cells were plated in 12-well plates at 80,000 cell/well (three wells for each condition to be tested; *n* = 3). Cells were transfected, as described above, 24 h after plating. In experiments involving autophagy blockage, 10 mM 3-MA was added to the cells for the last 48 h prior to protein extraction. Proteasome inhibition was performed by adding 10 μM MG132 treatment for the last 16 h (overnight treatment). Cells were harvested and centrifuged 5 min at 100×*g*, 72 h after plating, at 4 °C; the cell pellets were re-suspended in PBS (Sigma-Aldrich, P4417) added of the protease inhibitor cocktail (Sigma-Aldrich, P8340) and homogenized using slight sonication to lyse cells and nuclei as previously described (Crippa et al. 2016a; Crippa et al. 2016b; Giorgetti et al. 2015; Rusmini et al. 2013). Total proteins were determined with the bicinchoninic acid method (BCA assay; Euroclone, EMP014500).

Western blot (WB) was performed on 10% SDS–polyacrylamide gel electrophoresis loading 20 μg of total protein extracts. Samples were then electro-transferred to nitrocellulose membrane (Bio-Rad 1620115) using a trans-Blot apparatus (Mini Trans-Blot Cell; Bio-Rad Laboratories). The membranes were treated with a blocking solution containing 5% nonfat dried milk powder (Euroclone, EMR180500) in Tris-buffered saline with Tween 20 (0.01%) (TBS-T; Tris base 20 mM, NaCl 140 mM, pH 7.6) for 1 h and then incubated with one of the following primary antibodies: (a) mouse polyclonal ANTI-FLAG M2 (dilution 1:1000; Sigma-Aldrich, F1804) to detect DPRs; (b) mouse monoclonal anti-TUBA (dilution 1:4000; Sigma-Aldrich, T6199); and (c) homemade rabbit polyclonal anti-HSPB8 no. 25 (Carra et al. 2008a; Carra et al. 2005) (dilution 1:3000). Immunoreactivity was detected using the following secondary peroxidase-conjugated antibodies: goat anti-rabbit (dilution 1:10,000; Santa Cruz Biotechnology, sc-2004) and goat anti-mouse (dilution 1:10,000; Santa Cruz Biotechnology, sc-2005). Signals were revealed by chemiluminescence detection kit reagents (Clarity™ Western ECL Blotting Substrate; Bio-Rad, 170-5060). The same membranes were subsequently processed with different antibodies to detect the levels of different proteins in the same sample, after stripping for 20 min at room temperature (StripABlot; Euroclone, EMP100500). Filter retardation assay (FRA) was performed using a Bio-Dot SF Microfiltration Apparatus (Bio-Rad). Eight micrograms of the total proteins were filtered through a 0.2-μm cellulose acetate membrane (Whatman, 100404180). The membranes were probed as described for WB.

A ChemiDoc XRS System (Bio-Rad) was used for the image acquisition of WB and FRA. Optical density of samples assayed with WB or FRA was detected and analyzed using the Image Lab software (Bio-Rad). Statistical analyses have been performed using the relative optical densities defined as the ratio between the optical densities of each independent biological sample (*n* = 3) and the mean optical density of control samples.

### Statistical analysis

Data are presented as mean ± SD. Statistical analyses have been performed by using one-tailed unpaired Student’s *t* test to compare data between two groups and one-way ANOVA to compare more than two groups of data. When ANOVA resulted significant, we performed the Tukey’s post hoc test (and one-tailed unpaired Student’s *t* test when the variances between groups were highly different) (see figure legends for details) for multiple comparisons. Computations were done with the PRISM (ver. 6.0 h) software (GraphPad Software, La Jolla, CA, USA).

## Results

### Biochemical characterization of DPRs in immortalized motoneurons

**Fig. 1:**
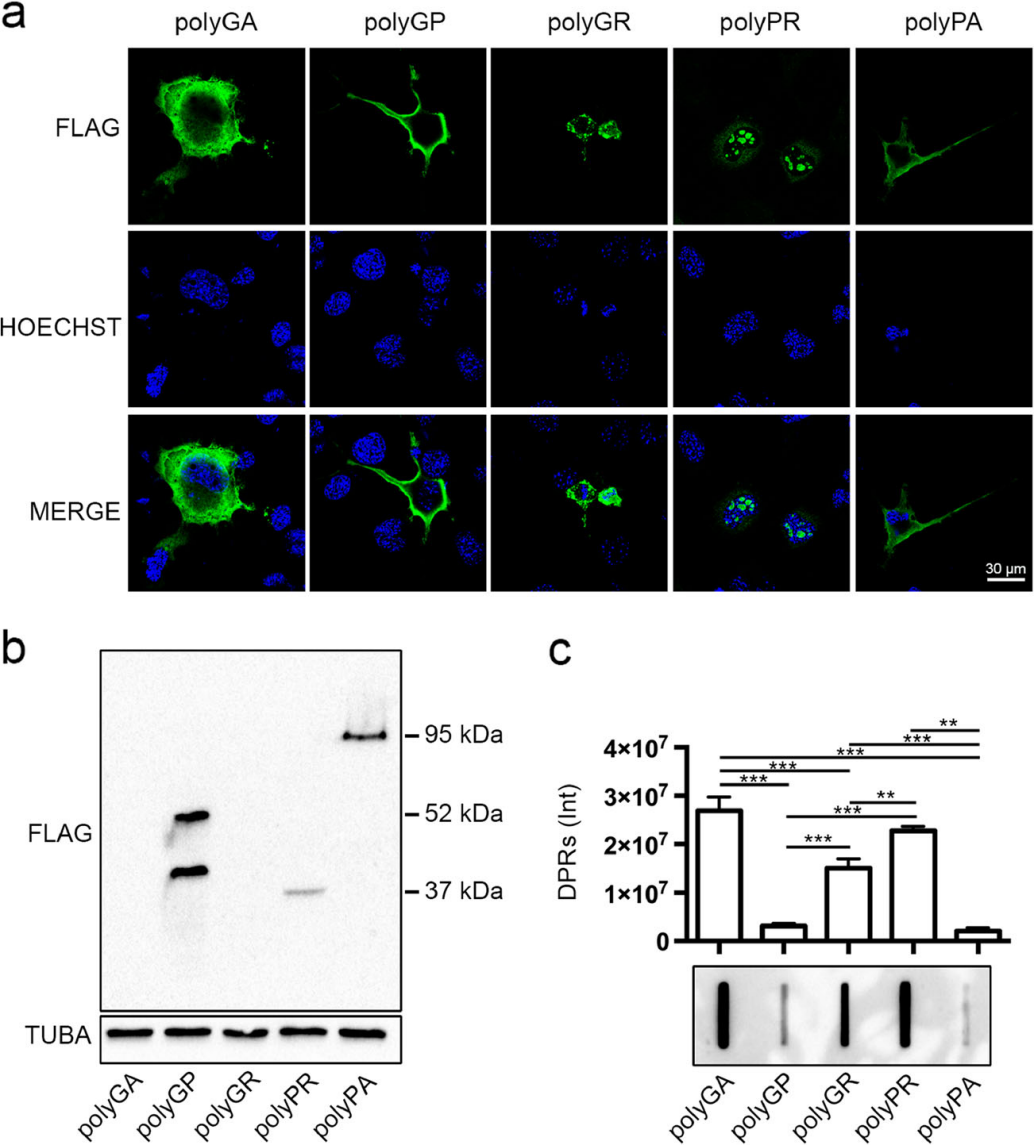
DPRs overexpression in NSC34 cells. **a** Confocal microscopy analysis of NSC34 cells shows DPRs localization (×63 magnification); *scale bars* 30 μm. **b**, **c** NSC34 cells were collected 48 h after transfection with FLAG-polyDPRs (*GA*, *GP*, *GR*, *PR*, *PA*). **b** WB shows DPR total levels. TUBA was used as loading control. **c** FRA shows PBS insoluble fraction of DPRs. Bar graph represents the FRA mean relative optical density computed over three independent biological samples for each condition (*n* = 3) ± SD (**p < 0.01, ***p < 0.001; one-way ANOVA followed by Tukey’s test)

**Fig. 2:**
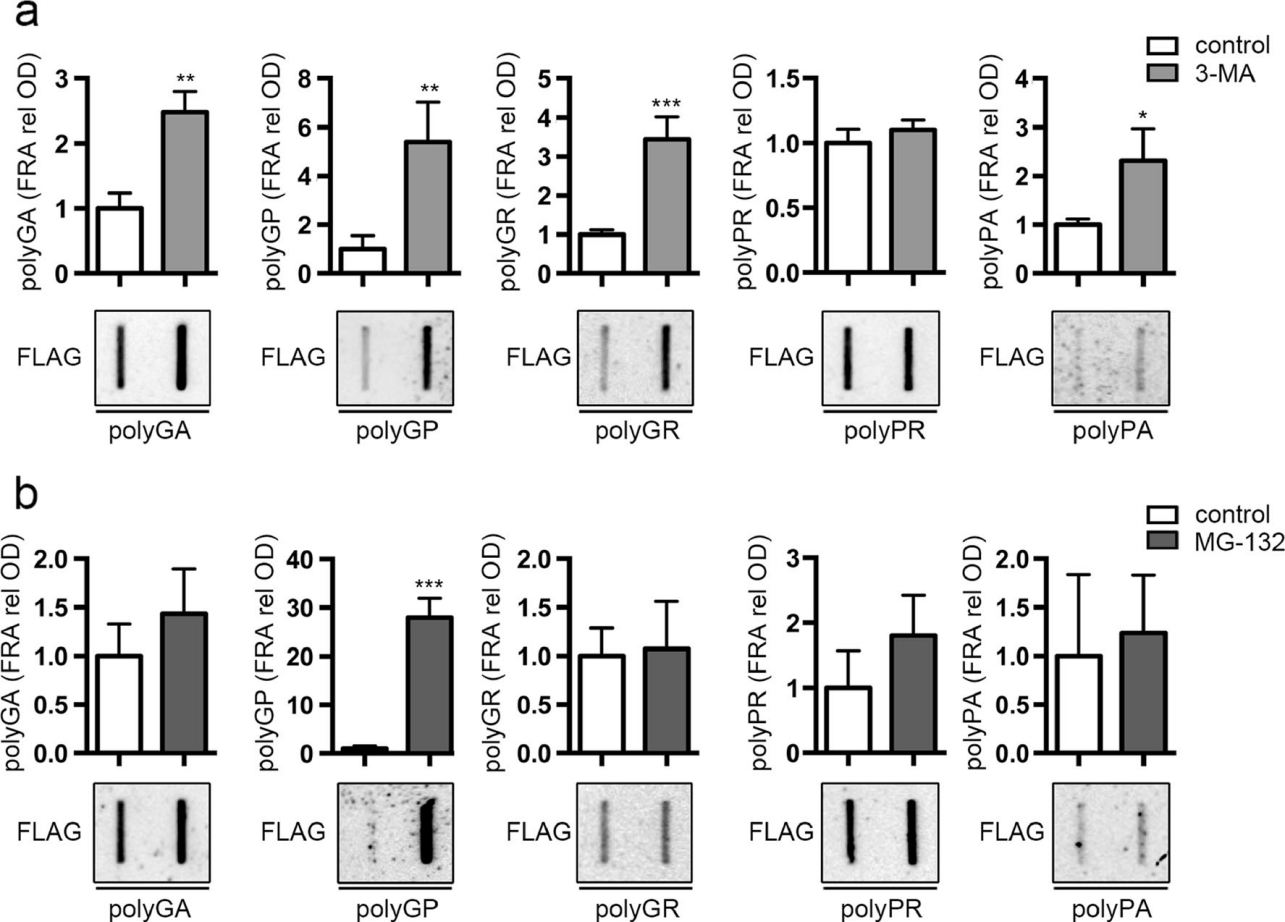
Effect of autophagy and proteasome inhibitors on the PBS insoluble levels of DPRs. NSC34 cells were collected 48 h after transfection with FLAG-polyDPRs (*GA*, *GP*, *GR*, *PR*, *PA*). **a** FRA shows PBS insoluble fraction of DPRs after 48 h of autophagy inhibition treatment with 10 mM 3-MA. Bar graph represents the FRA mean relative optical density computed over three independent biological samples for each condition (*n* = 3) ± SD (*p < 0.05, **p < 0.01, ***p < 0.001; Student’s *t* test). **b** FRA shows PBS insoluble fraction of DPRs after 16 h of proteasome inhibition treatment with 10 μM MG-132. Bar graph represents the FRA mean relative optical density computed over three independent biological samples for each condition (*n* = 3) ± SD (***p < 0.001; Student’s *t* test)

To evaluate the biochemical behavior of the DPRs deriving from RAN translation of the G4C2 of the *C9ORF72* transcript, we used artificial cDNAs expressing each single flagged DPR under the control of the CMV promoter. We kept neuronal cell growth and transfection conditions identical for all plasmids carrying the five different DPR-encoding sequences in order to ensure DPR identical expression. We initially tested in immortalized motoneurons the level of the five DPRs by immunofluorescence (IF), WB, and FRA using an anti-FLAG antibody. Fig. 1a illustrates the IF analysis, which revealed that the five DPRs have very different intracellular localization and aggregation propensity. In fact, polyGA was uniformly distributed into the entire cytoplasm of motoneuronal cell, with very few detectable small aggregates, while polyGP and polyPA mainly distributed in peripheral cell region possibly associated to the cell membranes. Only polyGR and polyPR were clearly detectable in aggregate form. The polyGR inclusions mainly localized in the cytoplasm, while the polyPR inclusions were confined into the cell nucleus. By analyzing DPR levels in WB, we found that their SDS-soluble monomeric forms considerably varied, being very high for the polyGP, moderate for the polyPA, low for polyPR, and absent for polyGR and polyGA. At longer exposure of the ECL-processed membrane from WB, a tiny band of polyGA appeared (not shown). Expression of the polyGP resulted in two proteins with different molecular weight (M.W.) (about 40 and 55 kDa), while that of polyPA was observed at an apparent M.W. of 95 kDa and polyPR was observed at an apparent M.W. of 37 kDa (Fig. 1b). It is well known that proteins that have a high tendency to form aggregates become insoluble and cannot be detected with conventional WB (Tebbenkamp and Borchelt 2009). These aggregated species are instead detected with FRA (Carra et al. 2008a; Crippa et al. 2010b; Rusmini et al. 2013). We then analyzed by FRA whether the DPR species that are not detected by conventional WB, such as polyGA, polyGR, and polyPR, would instead accumulate in the form of aggregated species. In line with our hypothesis, the highest amount of PBS-insoluble material was found in lysates of motoneurons expressing the polyGA, polyPR, and polyGR, which were undetected or only moderately detected by WB (Fig. 1c). Conversely, polyGP and polyPA were mainly accumulated in form of SDS-soluble species and only poorly accumulated in form of PBS-insoluble species in motoneurons (Fig. 1b, c). These data indicate the existence of an inverse correlation between solubility and accumulation into insoluble aggregates of the five DPRs. We thus analyzed whether the different biochemical properties of the DPRs may relate to their selective intracellular processing by the PQC system. We selectively blocked autophagy or proteasome with the inhibitors 3-MA and MG132 respectively. We focused on the total levels of insoluble materials, which correspond to the species that accumulate probably due to an inefficient removal by the PQC system. The data suggested that the various DPRs are differentially degraded by these two alternative systems. In fact, autophagy blockage performed using the inhibitor 3-MA resulted in a significant increased of the insoluble fractions of most DPRs, except for polyPR (Fig. 2a). The levels of insoluble polyGA were increased ca. 2.5-folds by autophagy blockage. A similar effect was observed for polyPA (which also contains the hydrophobic amino acid alanine), although its soluble and insoluble levels are very low compared to the other DPRs. Interestingly, these two DPRs have a very different biochemical behavior in basal condition, since polyGA has the highest tendency to aggregate, while polyPA poorly aggregates and mainly exists in PBS soluble forms (see Fig. 1b, c). Moreover, both insoluble polyGA and polyPA accumulate when autophagosome formation is inhibited; in basal condition, only the polyGA remains present in large insoluble amounts in motoneuronal cells; it is likely that these DPRs (but not the polyPA) might be partially resistant to clearance using the autophagosome pathway. The effects of 3-MA were even more robust on the DPRs containing glycine; in fact, polyGP and polyGR, we noted, respectively, ca. 5- and 3.5-fold increases when autophagosome formation was inhibited with 3-MA (Fig. 2a). Notably, while in basal condition (see Fig. 1c), polyGP poorly aggregates, polyGR forms large amounts of PBS-insoluble materials. Therefore, the presence of a proline instead of an arginine, together with the glycine, significantly modified the capability of these DPRs to be removed using autophagy. Instead, the clearance was almost complete for the polyGP and only moderate for the polyGR. Similar results were observed for the other DPR containing arginine. In contrast, the polyPR, which exists both in PBS-soluble and insoluble forms, apparently is not processed utilizing the autophagic system on our motoneuronal cells; in fact, we found very high levels of polyPR accumulating in basal condition (Fig. 1c), which were not modified by 3-MA (Fig. 2a). Collectively, these results suggest that autophagy participates to the clearance of DPRs, except for polyPR.

Concerning the contribution of the proteasome in the removal of DPRs, we found that only polyGP insoluble species were significantly accumulated following proteasome inhibition (Fig. 2b). Instead, the insoluble levels of the other four DPRs remained unchanged before and after MG132 treatment. In combination, these data show that in immortalized motoneurons, polyGP is the only DPR processed by both degradative systems, while the other mainly relies on functional autophagy.

### Upregulation of HSPB8 decreases the accumulation of DPRs

Previous studies, including ours, demonstrated that HSPB8 can recognize a number of misfolded proteins associated to neurodegenerative conditions (ARpolyQ, HTT, mutant SOD1, TDP-43 and its disease-associated fragments, A-beta, alpha-syn) (Bruinsma et al. 2011; Carra et al. 2008a; Crippa et al. 2010a; Crippa et al. 2010b; Giorgetti et al. 2015; Rusmini et al. 2013; Wilhelmus et al. 2006), and even proteins unrelated to NDs (Arndt et al. 2010; Chavez Zobel et al. 2003; Sanbe et al. 2009). HSPB8, acting in concert with the BAG3-HSP70 machinery, facilitates the autophagic removal of misfolded proteins. Our biochemical characterization showed that polyGA, polyGR, and polyPR mainly accumulate in the PBS-insoluble fraction; moreover, except for polyPR, whose levels are unchanged upon autophagy inhibition, the other four DPRs analyzed are all cleared by autophagy. Here, we analyzed whether HSPB8 can also recognize the RAN translated “aberrant” DPR species that cannot be considered “misfolded proteins” and normally do not exist in cells, facilitating their autophagy-mediated clearance.

**Fig. 3:**
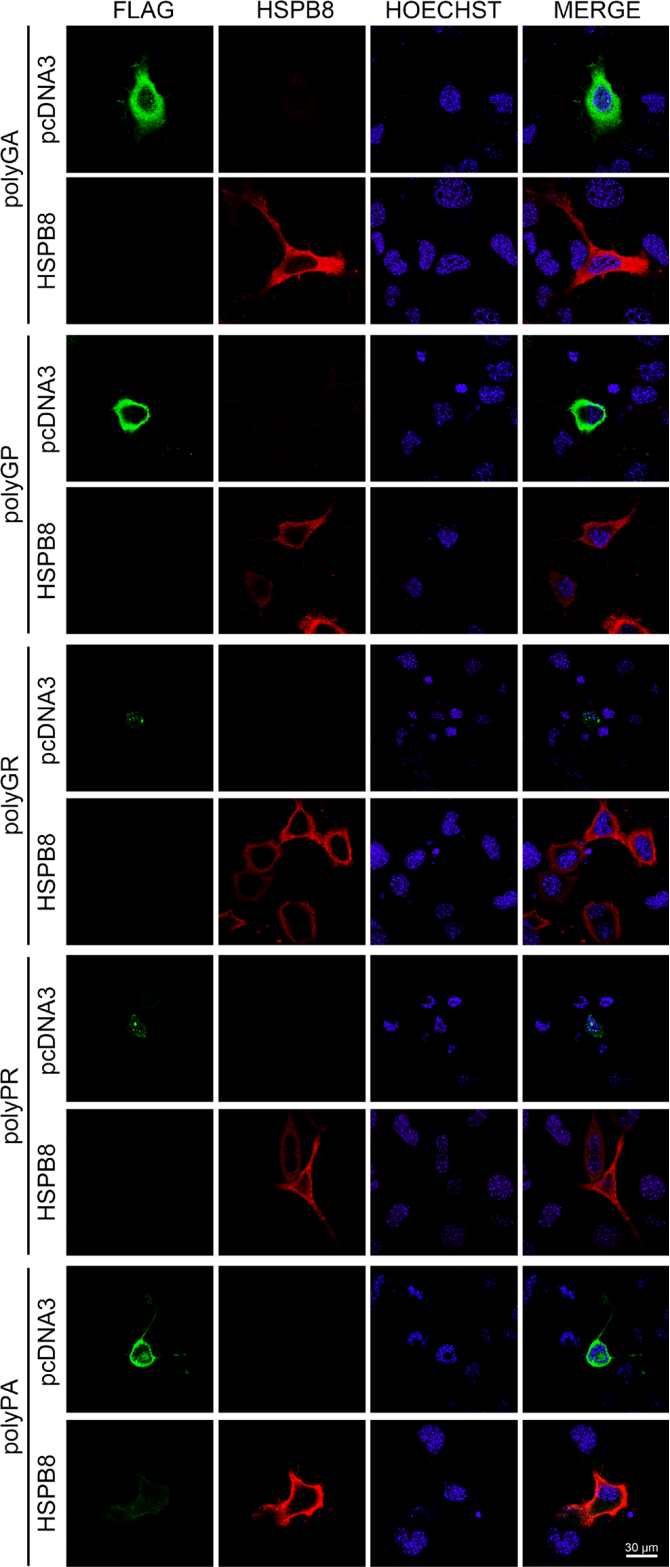
Effect of HSPB8 overexpression on DPRs distribution. NSC34 cells were fixed 48 h after transfection with FLAG-polyDPRs (*GA*, *GP*, *GR*, *PR*, *PA*) and pCI-HSPB8 or pcDNA3. IF shows DPRs distribution (×63 magnification); *scale bars* 30 μm

**Fig. 4:**
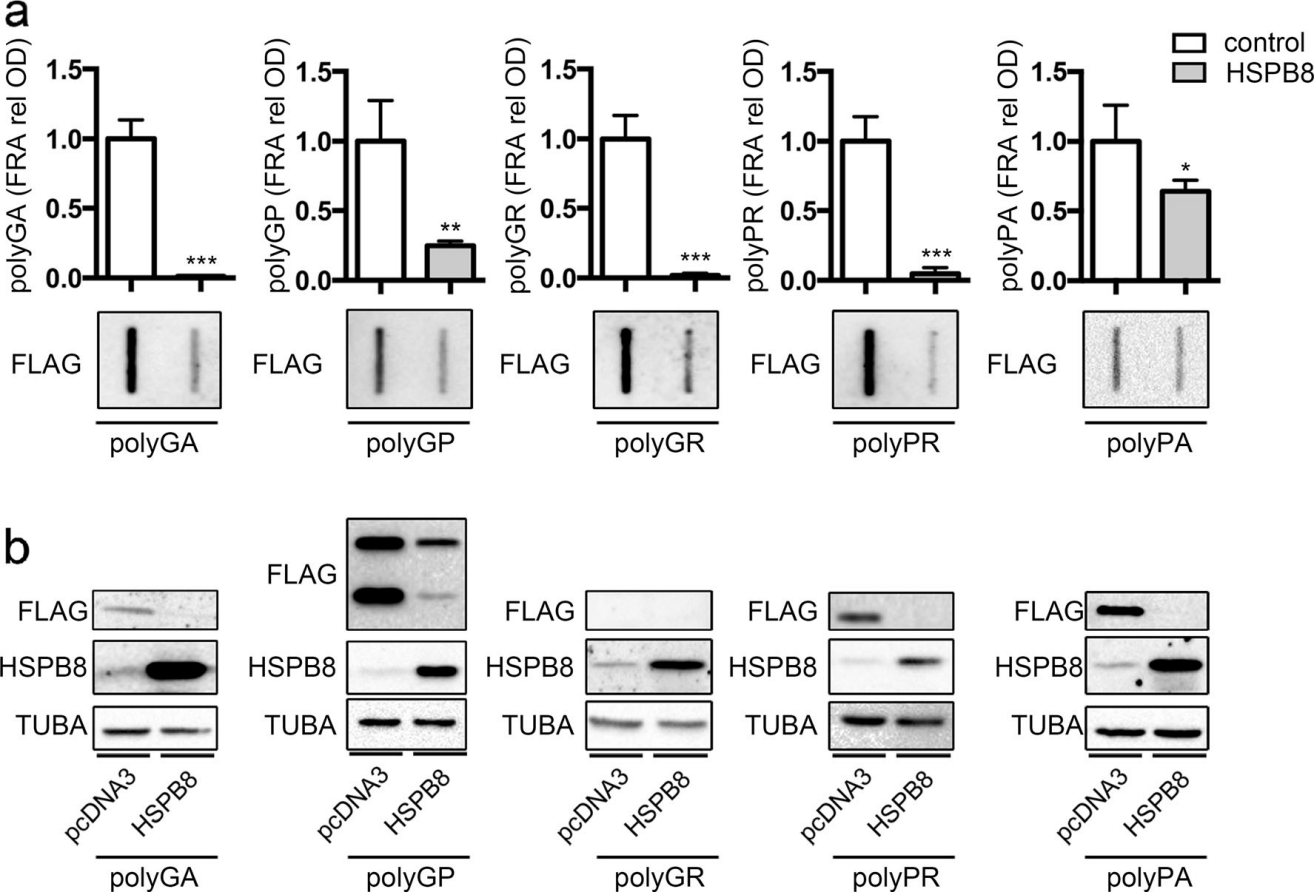
Effect of HSPB8 overexpression on DPRs levels. NSC34 cells were collected 48 h after transfection with FLAG-polyDPRs (*GA*, *GP*, *GR*, *PR*, *PA*) and pCI-HSPB8 or pCDNA3. **a** FRA shows PBS insoluble fraction of DPRs. Bar graph represents the FRA mean relative optical density computed over three independent biological samples for each condition (*n* = 3) ± SD (**p < 0.01, ***p < 0.001; Student’s *t* test). **b** WB shows DPR total levels; TUBA was used as loading control

To this purpose, we upregulated or silenced HSPB8 expression in immortalized motoneurons expressing each single DPR and we measured the variation of aggregates of insoluble material in IF and FRA, respectively. Figure 3 shows that HSPB8 overexpression fully removes all five detectable DPRs from cells, independently from their intracellular localization or aggregated status. In fact, both the soluble cytoplasmic polyGA and the two aggregated polyGR and polyPR species disappeared in cells overexpressing HSPB8. Also, the membrane associated polyGP and polyPA DPRs were undetectable in the presence of overexpressed HSPB8. In line with the IF analysis, the data obtained with FRA, reported in Fig. 4a, clearly demonstrated that increased levels of HSPB8 greatly reduced the accumulation of insoluble species formed by all DPRs. The effect of HSPB8 was particularly pronounced on polyGA, polyGR, and polyPR, for which the removal of insoluble DPR species was almost complete, even if these DPRs accumulated at very high levels in NSC34 cells (see Fig. 1). PolyGP and polyPA are already actively processed by autophagy in basal condition (see Figs. 1c and 2b); thus, the pro-autophagic facilitation exerted by HSPB8 was slightly less evident for these DPRs. Notably, HSPB8 also decreased the total PBS-soluble levels of the various DPRs, measured by WB, except for PBS-soluble polyGR that is almost undetectable (Fig. 4b).

**Fig. 5:**
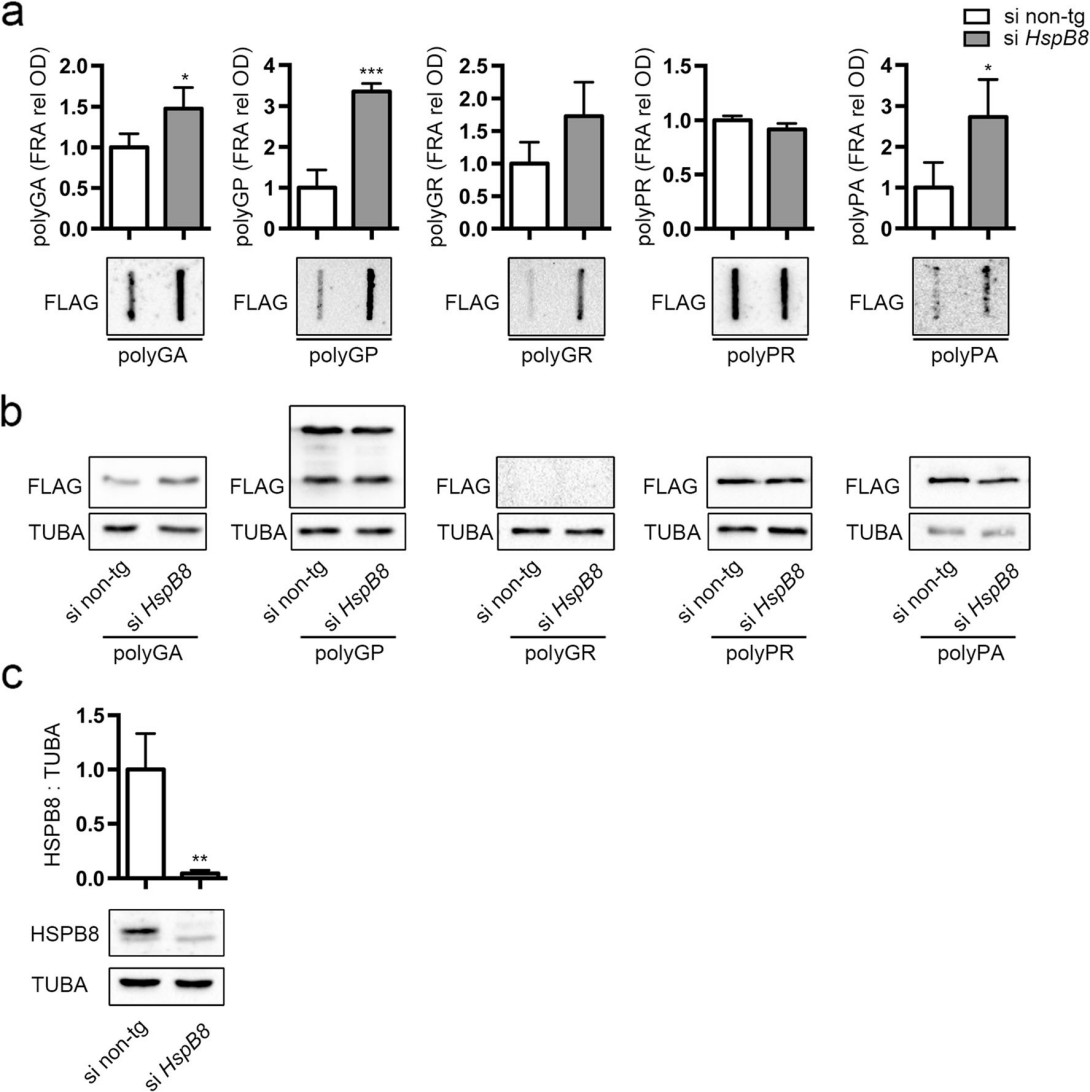
Effect of HSPB8 silencing on DPRs levels. NSC34 cells were transfected with non-target or *HspB8* siRNA and FLAG-polyDPRs (*GA*, *GP*, *GR*, *PR*, *PA*). **a** FRA shows PBS insoluble fraction of DPRs. Bar graph represents the FRA mean relative optical density computed over three independent biological samples for each condition (*n* = 3) ± SD (* = *p* < 0.05, *** = *p* < 0.001; Student’s *t* test). **b** WB shows DPRs total levels; TUBA was used as loading control. **c** WB shows HSPB8 levels; TUBA was used as loading control. Bar graph represents the HSPB8/TUBA ratio computed over three independent biological samples for each condition (*n* = 3) ± SD (** = *p* < 0.01; Student’s *t* test)

We then downregulated HSPB8 expression using a specific siRNA (Fig. 5c). The IF analysis indicated that HSPB8 silencing resulted in an increased number of DPR positive cells, without affecting distribution and localization in a measurable manner (data not shown), since IF is not quantitative to evaluate the effect of HSPB8. By measuring its effects on the levels of accumulation of PBS-soluble and insoluble DPR species we found that the levels of insoluble polyGA, polyGP, and polyPA (although the latter accumulating at lower levels) species formed in NSC34 cells were increased by the removal of endogenous HSPB8 (Fig. 5a). Insoluble polyGR species, which significantly increased after autophagy blockage (Fig. 2a), also tended to increase when HSPB8 is silenced, without reaching a statistical significance (Fig. 5a). Conversely, polyPR remained unchanged both after autophagy blockage (Fig. 2a) and HSPB8 dowregulation (Fig. 5a). It is likely that the endogenous HSPB8 levels are not sufficient to clear polyPR aggregates from motoneuronal cells via autophagy. However, exogenously expressed HSPB8 (at higher levels) greatly facilitated autophagic clearance of polyPR soluble and insoluble species (see Fig. 4). Thus, polyPR, which has a high tendency to aggregate (Fig. 1c), could readily accumulate in PBS-insoluble forms that are less efficiently degraded by autophagy. Increasing the expression of HSPB8 might keep polyPR in a state competent for clearance, as it occurs following overexpression of HSPB8 (Fig. 4). Notably, with HSPB8 silencing, we did not find an increase of the soluble levels of DPRs in WB (Fig. 5b), suggesting that the endogenous HSPB8 would act primarily on the insoluble DPR species. Instead, upon HSPB8 overexpression, both soluble and insoluble DPR species significantly decreased in immortalized motoneurons. In combination, these results suggest that overexpressed HSPB8 may keep all DPRs in a state competent for disposal by the cells, avoiding their aggregation.

## Discussion

In this study, we provide a biochemical characterization of five different RAN translated DPRs from the C9ORF72 transcript containing an expanded G4C2 stretch in immortalized motoneuronal cells. Since DPRs cannot be considered as classical misfolded proteins, but likely generate abnormal structures uncommon for neuronal cells, we also evaluated whether HSPB8, a chaperone able to enhance misfolded protein autophagic clearance, was active on the five DPRs.

We found that the five DPRs have a very different localization and propensity to accumulate in immortalized motoneurons. Formation of insoluble material was very low for the polyGP and polyPA DPRs, which seems to be processed via autophagy; instead, the three remaining DPRs analyzed showed a very high tendency to accumulate in form of insoluble species. Interestingly, the polyGP and polyPA are uncharged DPRs with a compact flexible coil structure (Lee et al. 2016). The solubility data that we obtained are generally in line with a previous report from Yamakawa et al. (2015), even if in this study, differences were reported by using alternative cell lines (N2a and 293T cells). Concerning the preferred pathway of degradation, this was not uniform for the five DPRs. Of all five DPRs, only the polyGP seems to be efficiently removed via the proteasome, while the others are apparently mainly degraded via autophagy, except for polyPR, which is not significantly affected by 3-MA treatment or HSPB8 depletion. Since polyPR inclusions are mostly detectable in the nucleus, it is possible that they cannot be cleared by autophagy, which is exclusively a cytoplasmic process. The fact that HSPB8 is active also on this DPR in basal condition suggests that the action of this chaperone may take place before polyPR nuclear import and aggregation. Despite their different localization, all DPRs clearance was enhanced by HSPB8. Instead, in N2a and 293T cells, also polyGR and polyPR can be cleared via this pathway (Yamakawa et al. 2015). In combination, these results suggest that different mammalian cell lines differentially use both degradative pathways to clear the DPRs. Although the proteasome or autophagy-mediated clearance of the five DPRs seems to vary based on the cell type used, at least in motoneuronal cells, the presence of proline only with glycine (in polyGP), and not with arginine or alanine (in polyPR and polyPA, respectively) correlates with the utilization of the proteasome pathway rather than the autophagic pathway for clearance. Whether it is the type of amino acid or rather the differential tendency to form oligomeric aggregation-prone species that influences the pathway that is mainly degrading one specific type of DPR will require future investigations.

Next, we tested the effects of upregulation and downregulation of HSPB8 on DPRs accumulation and clearance in motoneurons. The selection of HSPB8 was based on the following reasons: (1) as previously mentioned, HSPB8 was shown to enhance the autophagy-mediated clearance of a large variety of aggregation-prone proteins associated with motoneuron and neurodegenerative diseases (Carra et al. 2013; Crippa et al. 2013b; Rusmini et al. 2016); (2) HSPB8 is induced in the motor neuron surviving at late stage of disease in the spinal cord of SOD1 ALS mice, as well as in patients affected by ALS (Crippa et al. 2010a; Crippa et al. 2010b); and (3) mutations in the HSPB8 gene lead to motor neuropathy (Evgrafov et al. 2004; Ikeda et al. 2009; Irobi et al. 2004; Kwok et al. 2011; Tang et al. 2005; Ghaoui et al. 2016). Altogether, these findings support the interpretation that deregulation in the expression levels of HSPB8 might render motoneurons more vulnerable to proteotoxic insults, while its induction might protect against the toxicity exerted by aggregation-prone species, including DPRs, which accumulate with high frequency in ALS and FTD patients (Al-Sarraj et al. 2011). We found that HSPB8 overexpression significantly and robustly counteracts the accumulation of insoluble species of all five DPRs. Exogenously expressed HSPB8 decreased the accumulation of both aggregating species and the total soluble protein levels detectable in WB. We noticed that HSPB8 is particular active in decreasing the levels of those DPRs characterized by an high propensity to form insoluble species (polyGA, polyGR, and polyPR), including polyGA, which is the one showing the strongest propensity to generate Congo red or thioflavin T positive amyloidogenic fibrils (May et al. 2014; Chang et al. 2016) with a parallel β-sheet structure like those of the beta-amyloid (Chang et al. 2016; Edbauer and Haass 2016). Notably, the polyGA has been reported to be less toxic (Freibaum et al. 2015; Lee et al. 2016; Mizielinska et al. 2014; Wen et al. 2014) than polyGR and polyPR (two highly charged and polar DPRs, since they contain the arginine amino acid), which are thought to be the most toxic at very low concentrations.

When we silenced the endogenous expression of HSPB8, we found a significant increase of insoluble species of three out of five DPRs (polyGA, polyGP, and polyPA); the effect on polyPA was significant, but the total levels of accumulated material remain low. Conversely, polyGR tended to increase, even if the variation was not statistically relevant. Instead, polyPR levels remained unaffected upon depletion of HSPB8. Interestingly, the downregulation of endogenous HSPB8 has no effect on the overall soluble levels of the DPRs, suggesting that in resting cells, HSPB8 would only target aggregating species, favoring their autophagy-mediated clearance. This interpretation is supported by the observation that the only DPR that is poorly processed by autophagy in motoneurons, namely polyPR, is also the only one that is not affected by the depletion of HSPB8. Altogether, the study here reported has shown that HSPB8 recognizes and facilitates clearance of insoluble species of these peculiar DPRs, whose structures may not reflect those typically formed by “classical” misfolded proteins responsible for neurodegenerative diseases. It remains to be determined how HSPB8 recognizes these structures, and whether it acts also on a mixed pool of the five DPRs or rather it preferentially binds to the more aggregation-prone species.
